# Educational program for patients with type-1 diabetes mellitus receiving free monthly supplies of insulin improves knowledge and attitude, but not adherence

**DOI:** 10.4103/0973-3930.62602

**Published:** 2010

**Authors:** Abdus Salam

**Affiliations:** Department of Medical Education, Universiti Kebangsaan, Malaysia

Dear Sir,

I have read with interest the article titled, ‘Educational program for patients with type-1 diabetes mellitus receiving free monthly supplies of insulin improves knowledge and attitude, but not adherence,’ Vimalavathini *et al*.[1] The authors mention in the second paragraph of introduction, “When a patient does not respond to an appropriately prescribed medicine, the reasons could be drug or patient-related factors”. I agree, but at the same time I want to add that, the reasons could also be communication-related factors. The authors already mention in the third paragraph that, “Planned interventional education programs have shown to provide a positive impact on improving the KAP scores in diabetic patients”.

Education is a broad concept, which encompasses both teaching and learning. Evidence-based studies show that doctors' interpersonal and communication skills have a significant impact on improved health outcomes.[2–4] To provide comprehensive care, many key qualities are essential, which include the ability to communicate effectively with the patient, act in a professional manner, cultivate an awareness of one's own values and prejudices, and provide care with an understanding of the cultural and spiritual dimensions of the patient's life.[5]

Quality drugs, discipline, and diet (3D) are the principles of diabetic management. However, patients' adherence, compliance with medication, and disease outcome are closely associated with the quality of communication and a planned interventional education program.
